# Relationship between Funding Source and Conclusion among Nutrition-Related Scientific Articles

**DOI:** 10.1371/journal.pmed.0040005

**Published:** 2007-01-09

**Authors:** Lenard I Lesser, Cara B Ebbeling, Merrill Goozner, David Wypij, David S Ludwig

**Affiliations:** 1 Department of Medicine, Children's Hospital, Boston, Massachusetts, United States of America; 2 The Center for Science in the Public Interest, Washington, D. C., United States of America; 3 Department of Cardiology, Children's Hospital, Boston, Massachusetts, United States of America; 4 Clinical Research Program, Children's Hospital, Boston, Massachusetts, United States of America; Vrije Universiteit Amsterdam, The Netherlands

## Abstract

**Background:**

Industrial support of biomedical research may bias scientific conclusions, as demonstrated by recent analyses of pharmaceutical studies. However, this issue has not been systematically examined in the area of nutrition research. The purpose of this study is to characterize financial sponsorship of scientific articles addressing the health effects of three commonly consumed beverages, and to determine how sponsorship affects published conclusions.

**Methods and Findings:**

Medline searches of worldwide literature were used to identify three article types (interventional studies, observational studies, and scientific reviews) about soft drinks, juice, and milk published between 1 January, 1999 and 31 December, 2003. Financial sponsorship and article conclusions were classified by independent groups of coinvestigators. The relationship between sponsorship and conclusions was explored by exact tests and regression analyses, controlling for covariates. 206 articles were included in the study, of which 111 declared financial sponsorship. Of these, 22% had all industry funding, 47% had no industry funding, and 32% had mixed funding. Funding source was significantly related to conclusions when considering all article types (*p* = 0.037). For interventional studies, the proportion with unfavorable conclusions was 0% for all industry funding versus 37% for no industry funding (*p* = 0.009). The odds ratio of a favorable versus unfavorable conclusion was 7.61 (95% confidence interval 1.27 to 45.73), comparing articles with all industry funding to no industry funding.

**Conclusions:**

Industry funding of nutrition-related scientific articles may bias conclusions in favor of sponsors' products, with potentially significant implications for public health.

## Abstract 2

In 111 scientific articles on nonalcoholic beverages, articles with all industry funding were more than 7 times more likely to have favorable conclusions compared with articles with no industry funding.

## Editors' Summary

**Background.:**

Much of the money available for doing medical research comes from companies, as opposed to government agencies or charities. There is some evidence that when a research study is sponsored by an organization that has a financial interest in the outcome, the study is more likely to produce results that favor the funder (this is called “sponsorship bias”). This phenomenon is worrying, because if our knowledge about effectiveness and safety of medicines is based on biased findings, patients could suffer. However, it is not clear whether sponsorship bias extends beyond research into drugs, but also affects other types of research that is in the public interest. For example, research into the health benefits, or otherwise, of different types of food and drink may affect government guidelines, regulations, and the behavior patterns of members of the public. Were sponsorship bias also to exist in this area of research, the health of the wider public could be affected.

**Why Was This Study Done?:**

There is not a great deal of evidence about whether sponsorship bias affects nutritional research (scientific studies that look at the relationship between food and/or drink, and health or disease states). Therefore, the group of researchers here set out to collect information from published nutritional research papers, to see if the type of sponsorship for the research studies was in any way linked with whether the main conclusions were favorable or unfavorable to the sponsor.

**What Did the Researchers Do and Find?:**

The research study reported here used the scientific literature as a source of data. The researchers chose to examine one particular area of nutrition (nonalcoholic drinks including soft drinks, juices, and milk), so that their investigation would not be affected too much by variability between the different types of nutritional research. Using literature searches, the researchers identified all original research and scientific review articles published between January 1999 and December 2003 that examined soft drinks, juices, and milk; described research carried out in humans; and at the same time drew conclusions relevant to health or disease. Then, information from each published article was categorized: the conclusions were coded as either favorable, unfavorable, or neutral in relation to the health effects of the products being studied, and the article's funding was coded as either all industry (ie, food/drinks companies), no industry, or mixed. 206 published articles were analyzed and only 54% declared funding. The researchers found that, overall, there was a strong association between the type of funding available for these articles and the conclusions that were drawn. Articles sponsored exclusively by food/drinks companies were four to eight times more likely to have conclusions favorable to the financial interests of the sponsoring company than articles which were not sponsored by food or drinks companies.

**What Do These Findings Mean?:**

These findings suggest that a high potential for bias exists in research into the health benefits or harms of nonalcoholic drinks. It is not clear from this research study why or how this bias comes about, but there are many different mechanisms that might cause it. The researchers suggest that certain initiatives might help to reduce bias, for example, increasing independent funding of nutrition research.

**Additional Information.:**

Please access these Web sites via the online version of this summary at http://dx.doi.org/doi:10.1371/journal.pmed.0040005.

- Conflict of Interest definition from Wikipedia (Wikipedia is an internet encyclopedia that anyone can edit)
- The International Committee of Medical Journal Editors provides standard guidelines for practices at medical journals, including a section on sponsorship, authorship, and accountability
- The Committee on Publication Ethics is a forum for journal editors to discuss issues related to the integrity of the scientific record, and it provides guidelines for editors and case studies for reference
- The Good Publication Practice guidelines outline standards for responsible publication of research sponsored by pharmaceutical companies

## Introduction

The extent of industrial funding for pharmaceutical research, and its implications for public health, have been extensively considered in recent years. Moses et al. reported that pharmaceutical firms provided 30% of the almost $100 billion spent on biomedical research in the United States in 2004 [1]. These expenditures raise concerns about the integrity of pharmaceutical research [2]. A meta-analysis by Bekelman et al. of 37 original quantitative studies of bias in pharmaceutical research found significant association between industry sponsorship and pro-industry conclusions (odds ratio [OR] 3.6; 95% confidence interval [CI], 2.63 to 4.91) [3].

In contrast, little information is available regarding the prevalence or impact of funding by the food industry on nutrition research. Whereas bias in pharmaceutical research could have an adverse effect on the health of millions of individuals who take medications, bias in nutrition research could have an adverse effect on the health of everyone. Findings of nutrition research influence the formulation of governmental and professional dietary guidelines, the design of public health interventions, and regulation of food product health claims. In addition, these findings may receive widespread publicity in the popular media, directly affecting consumer behavior.

Nestle examined a convenience sample of 11 studies from food, beverage, or supplement companies, reporting that “it [was] difficult to find studies that did not come to conclusions favoring the sponsor's commercial interest” [4]. Levine et al. found that authors taking a supportive compared to a critical or neutral position on the fat substitute olestra were much more likely to have a financial relationship with the manufacturer (80% versus 11% or 21%, respectively; *p* < 0.001) [5]. However, a systematic investigation of bias in nutrition research has not been conducted.

The aim of this study was to examine financial sponsorship of nutrition-related scientific articles, and whether sponsorship affects published conclusions. We hypothesized that scientific articles funded exclusively by the food industry or affiliated organizations would be more likely to have favorable conclusions than articles without industry-associated sponsorship. In 2003, approximately 10,000 nutrition-related scientific articles were published on many foods and nutrients, examining a variety of endpoints relating to numerous health states, and employing widely varying study designs (Medline literature search using the terms “nutrition” or “food” or “beverage,” limited to 2003, conducted on 15 March 2006). To avoid the methodological challenges arising from such great heterogeneity, we chose to focus our investigation on soft drinks, juices, and milk. The health risks and benefits of these three beverages have been the subject of much recent controversy, and the beverage industry is large and highly profitable, arguably creating an environment in which scientific bias might occur.

## Methods

### Design Overview

To avoid potential bias in our study design, coinvestigators independently selected articles for inclusion, analyzed article conclusions, and examined financial sponsors. Article conclusions were classified as “favorable,” “neutral,” or “unfavorable” by two coinvestigators who had no knowledge of financial sponsors. Funding source was classified as “all industry,” “no industry,” “mixed,” or “not stated” by another coinvestigator who had no knowledge of article conclusions. In addition, relationship of sponsors to the beverage being studied was characterized according to whether a favorable finding would have “benefit,” “no relationship,” or “antagonism” to apparent financial interests. Then, associations between funding source and article conclusion were calculated, with adjustment for relevant covariates in some models.

### Selection of Articles

We aimed to take the broadest view of the literature within the area of beverages and health, and therefore included a range of article types in the categories of interventional studies, observational studies, and scientific reviews. Scientific reviews—but not commentaries, editorials, or letters—were included because these articles are abundant, often cited, and potentially influential, and generally presumed to be objective. (However, we found very few scientific reviews that declared a source of funding; therefore, the numerical contribution of these articles to analyses of potential bias was small. We also conducted an analysis with scientific reviews excluded, focusing on only interventional studies.)

Articles included in this study were initially identified by the study coordinator (LIL) using OVID-Medline literature searches. The literature searches were designed in consultation with a medical librarian to have high sensitivity and intermediate specificity for inclusion criteria using the following terms to identify articles focusing on the beverages of interest: soft drinks, carbonated beverages; fruit juice, apple juice, orange juice, prune juice, cranberry juice, grapefruit juice, grape juice, guava juice, pear juice, pineapple juice, vegetable juice, carrot juice, tomato juice; milk. Additional terms for health and disease states of interest were included in the searches.

We used six inclusion criteria for study articles: (1) The topic relates directly to soft drinks, juices, or milk, or an inherent component of one of these beverages (e.g., calcium in milk). (2) At least one main endpoint relates directly to health, disease, or a disease marker. For example, an article demonstrating a health benefit of antioxidants in juice would be included, whereas an article describing manufacturing techniques to maximize antioxidant concentrations in juice would be excluded. (3) The article involves or considers research with humans or clinical materials derived from humans. (4) Conclusions relate directly to the beverage under study. For example, an article examining the effect of dietary calcium on bone mineral density would be included only if implications to the health effects of milk consumption are stated explicitly. (5) The article is classified as an interventional study, an observational study, or a scientific review according to standardized criteria listed below (see Assessment of Covariates). Articles in the categories of commentaries, editorials, letters, and miscellaneous were excluded. (6) The article was published in the 5-y period between 1 January 1999 and 31 December 2003.

Articles identified by literature search were then examined individually by the study coordinator and removed if any inclusion criteria were not met. Several additional articles were removed from the study for the following reasons: article was coauthored by one of the coinvestigators involved in this study (to avoid potential bias); one of the coinvestigators involved in classifying article conclusions had previous knowledge of the article's sponsorship (also to avoid potential bias): or both coinvestigators involved in classifying article conclusions determined that all inclusion criteria were not met. (A list of included articles is available from the authors upon request.)

### Classification of Article Conclusions

The study coordinator provided two coinvestigators (CBE and DSL) with each article's abstract and discussion/conclusion section (as available). The coinvestigators were given no information relating to the identity of the article (e.g., journal, title, authors) or to financial sponsorship. When electronic documents were available, a simple text file was utilized. For articles without electronic versions, photocopies were made that excluded or obscured any identifying information. The coinvestigators classified article conclusions independently and then met to resolve discrepancies, using the categories outlined below.

Favorable—if both coinvestigators agreed that: (1) the conclusions suggested beneficial health effects or absence of expected adverse health effects, and (2) no statements were made that cast the product in a negative light.

Unfavorable—if both coinvestigators agreed that: (1) the conclusions suggested adverse health effects or absence of expected beneficial health effects, and (2) no statements were made that cast the product in a positive light.

Neutral—if the coinvestigators agreed that the conclusions were neither favorable nor unfavorable, or if the coinvestigators could not agree on classification.

### Characterization of Financial Sponsorship

The study coordinator examined each article (and supplemental material, if relevant) in its entirety for information about financial sponsorship. A coinvestigator (MG) was given a list of all sponsors of each article linked to the type of beverage under study (soft drinks, juice, or milk). The coinvestigator was given no further information relating to the identity of the article (e.g., journal, title, authors) or to its methods or results.

The coinvestigator used generally available information, obtained in part by Internet searches, to characterize each sponsor as: (1) industry—including for profit and nonprofit affiliations (e.g., US National Dairy Council), (2) industry-associated—including governmental agencies that work with industry to promote consumption of specific foods or commodities (e.g., US Department of Agriculture), (3) nonindustry—including governmental agencies with no industry association (e.g., US National Institutes of Health), university, and independent foundations, philanthropies, and other nonprofit organizations, and (4) unknown. Funding source was then classified for each article as outlined below.

All industry—if all sponsors were classified as category (1) above.

No industry—if all sponsors were classified as category (3) above.

Mixed—if any sponsor was classified as category (2) or (4) above, or if the article had sponsors that were classified into more than one category.

We considered the possibility that an industry sponsor might fund a study or scientific review examining a competitor's product. For example, milk and soft drink consumption are reciprocally related among children [6]; thus, a negative conclusion relating to soft drinks would arguably be advantageous to a dairy-affiliated organization. For this reason, the relationship of a financial sponsor to the beverage under study was characterized as outlined below.

Benefit—if a positive finding appeared to be in its commercial interest.

Antagonism—if a negative finding appeared to be in its commercial interest.

No relationship—if the sponsor appeared to have no commercial interest at stake.

Unknown—if commercial interest could not be determined.

Sponsors with no association or affiliation with the food industry (e.g., government, university, independent nonprofit) were characterized as “no relationship.” No articles in the “all industry” category had sponsorship that was characterized as “no relationship” or as “unknown,” nor did any have multiple sponsors in different categories. Thus, each “all industry” article could be subcategorized as “benefit” or “antagonism.”

### Assessment of Covariates

Three covariates were examined: publication year (available from Medline), article type, and potential author conflict.

Article type was classified according to the following definitions: interventional study—if humans consumed, or if human tissue was exposed to, a food or food component with the intention of measuring a biological response; observational study—if data were collected on participants without the intervention of the investigators; and scientific review—if no original data were reported and if published research was analyzed in a systematic fashion.

Potential author conflicts for each article were identified if an explicit statement to this effect was made in the article about any author; or if a coinvestigator (MG) determined that the declared affiliation for any author might benefit from a positive conclusion relating to the beverage under consideration.

### Statistical Treatment

To evaluate changes over time in the percentage of articles with declared funding, we used the Mantel-Haenszel chi-square test for trend and exact binomial 95% CIs.

For analyses of the relationship between conclusion and funding source, we focused on the most discrete categories of funding: all industry—benefit, no industry, and all industry—antagonism. Studies with mixed funding were excluded because they represent a heterogeneous group, with different proportions of industry funding, potentially obscuring underlying relationships. Studies with no listed funding were also excluded from these analyses.

We evaluated the association between article conclusion (favorable, neutral, and unfavorable) and funding source using an exact linear-by-linear association test, pooling all article types. When evaluating this association for only interventional studies, we used Fisher's exact test, collapsing articles with favorable and neutral conclusions.

Using logistic regression analysis, we calculated ORs of conclusions for all industry compared to no industry funding. We computed two sets of ORs, one collapsing articles with a favorable or neutral conclusion and the other eliminating those with a neutral conclusion. Adjusted analyses controlled for relevant covariates, including publication year, beverage type, and potential author conflict of interests. One all industry—antagonism article was categorized as unfavorable, a situation in which the sponsor was perceived to benefit from a negative conclusion about a competitor's product (see above). Therefore, we considered this article as favorable to the sponsor's interests, and reclassified it as such for the purpose of calculating ORs, per a priori hypothesis.

We used *p* < 0.05 (two-tailed) as a criterion for statistical significance. Computations were performed using software packages (SAS Institute, http://www.sas.com; Cytel, http://www.cytel.com; Stata Corporation, http://www.stata.com).

### Role of the Funding Source

The funders of the study had no role in study design, data collection, data analysis, data interpretation, or writing of the report. The corresponding author had full access to all the data in the study and had final responsibility for the decision to submit for publication.

## Results

**Figure 1:**
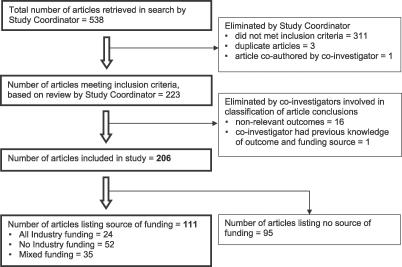
Flow Diagram for Inclusion of Articles in the Study

**Figure 2:**
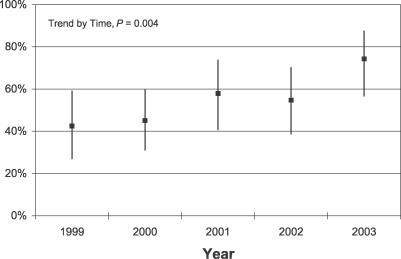
Percentage of Articles with Disclosed Funding by Publication Year (*n* = 206) Bars extend to the confidence limits of the exact binomial 95% CI. (Overall, funding declared for 62% of interventional studies, 67% of observational studies, and 19% of scientific reviews.)

Figure 1 depicts the flow diagram for inclusion of articles in the study. A total of 538 articles were retrieved in the searches, of which 332 were excluded. Descriptive data for the remaining 206 articles are presented in Table 1. Financial sponsorship was declared in 111 articles (54%). As shown in Figure 2, the proportion of articles disclosing sponsorship increased significantly from 1999 to 2003 (*p* for trend = 0.004). Considering all years together, 62% of interventional articles, 67% of observational articles, and 19% of scientific reviews indicated a funding source. Of those that reported sponsorship, 22% had all industry support, 47% had no industry support, and 32% had mixed funding.

**Table 1:**
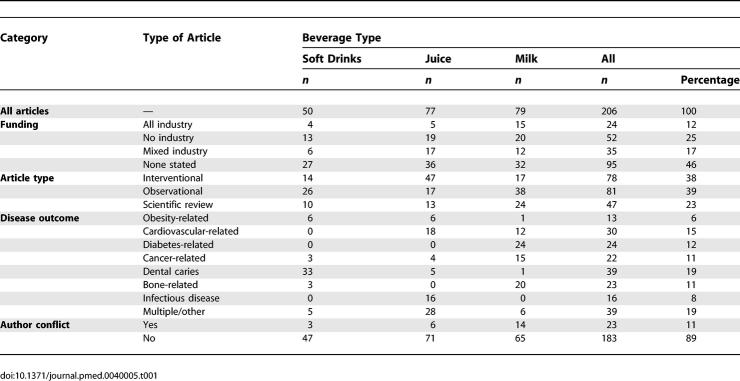
General Descriptive Data of Articles Included in Study (*n* = 206)

Overall, article conclusion was significantly related to funding source, as shown in Table 2 (*p* = 0.037). Among interventional studies, those with all industry compared to no industry support were much less likely to have an unfavorable conclusion (0% versus 37%; *p* = 0.009) (Table 3).

**Table 2:**
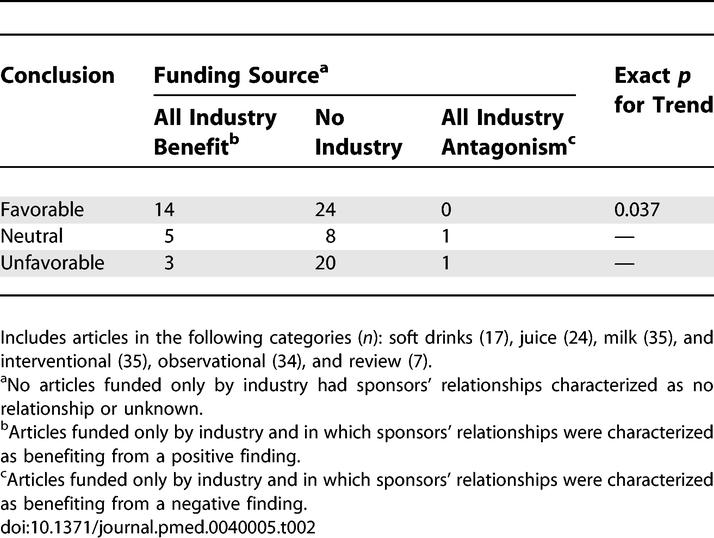
Relationship between Funding Source and Article Conclusions (*n* = 76)

**Table 3:**
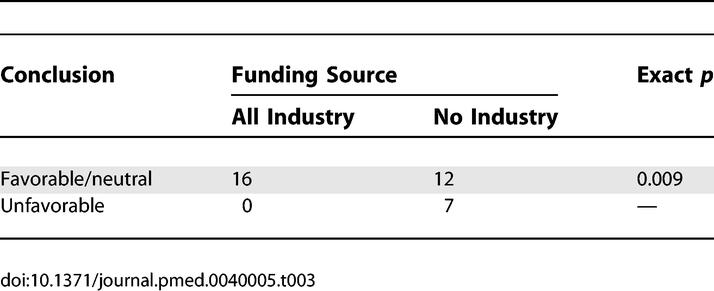
Relationship between Funding Source and Conclusions of Interventional Studies (*n* = 35)

The OR for a favorable or neutral versus unfavorable conclusion, comparing all industry to no industry support, was 6.18 (95% CI, 1.20 to 31.92) after adjustment for relevant covariates (Table 4). For the same comparison eliminating neutral articles, the OR was 7.61 (95% CI, 1.27 to 45.73).

**Table 4:**
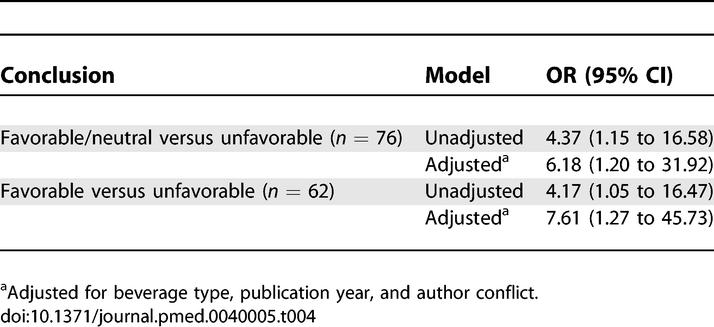
Odds Ratios for Conclusions of Articles with All Industry Compared to No Industry Support

## Discussion

The main finding of this study is that scientific articles about commonly consumed beverages funded entirely by industry were approximately four to eight times more likely to be favorable to the financial interests of the sponsors than articles without industry-related funding. Of particular interest, none of the interventional studies with all industry support had an unfavorable conclusion. Our study also documented industry sponsorship was very common during the study period, indicating considerable potential for introduction of bias into the biomedical literature. In view of the high consumption rates of these beverages, especially among children, the public health implications of this bias could be substantial.

The strengths of this study include a systematic method for identifying articles using objective criteria, a multi-level review process in which separate groups of coinvestigators collected data about conclusions and sponsorships without access to information that might bias judgment, and examination of a dynamic period in financial disclosure, during which the proportion of articles indicating funding had increased.

The primary limitation of the study is that we included only articles pertaining to three beverage categories; thus, the generalizability of our findings to other areas of nutrition is unknown. Because the articles varied greatly in approach, we were unable to collect meaningful information about factors that might reveal specific causes of bias, such as study quality. In addition, we made no attempt to obtain independent information about study sponsorship beyond that declared in the articles, nor did we assess other types of financial support, such as provision of supplies. Inaccurate or incomplete information about financial sponsorship may have caused us to underestimate the true magnitude of the relationship between conclusion and funding source. During the study period, declaration of financial support remained incomplete, especially for scientific reviews.

Studies of research supported by the pharmaceutical industry have suggested several ways that bias might be introduced into clinical trials [7–9], and some of these might apply to nutrition research. We speculate that our findings may relate to one or more of the following possibilities: (1) Industrial sponsors may fund only those studies that they believe will present their products in a favorable light, or their competitors' products in an unfavorable light. In support of this possibility, all studies funded entirely by industry were characterized as “benefit” or “antagonism” with regard to the product under study (none were characterized as “no relationship”). That is, industrial organizations do not seem to sponsor articles about products in which they have no financial interest. (2) Investigators might formulate hypotheses, design studies, or analyze data in ways that are consistent with the financial interests of their industrial sponsors. (3) Industrial sponsors or investigators may choose to delay or not publish findings that have negative implications to the sponsor's product. (4) Authors of scientific reviews may search and interpret the literature selectively, in ways consistent with the sponsor's interests. (5) Scientific reviews arising from industry-supported scientific symposia, often published as journal supplements, may over- or under-represent certain viewpoints, if presenters whose opinions conflict with the sponsor's financial interests are not invited to participate.

Some might question the significance of our findings, arguing that no investigator could ever be completely free of bias. We agree that financial conflict is not the only cause of bias. Indeed, the hallmark of modern scientific method, the a priori hypothesis, indicates a preconceived notion of how an experiment will unfold. Moreover, long-standing scientific viewpoints, career considerations, and even political opinions might color study design or interpretation. However, these types of individual bias tend to cancel themselves out among large groups of scientists over the long term. While one investigator's career may rise on a cherished theory, another's may rise by debunking that theory. We contend that financial conflict of interest is qualitatively different, producing selective bias that acts consistently in one direction over time. This contention receives support from a study by Kjaergard and Als-Nielsen, who found that authors' financial competing interests, but not their other competing interests, biased the conclusions of clinical trials [8].

If the findings of our study are supported by additional research, several ways to reduce bias among nutrition articles could be considered, including voluntary refusal by scientists to accept industrial support, regulations by academic institutions ensuring that publication decisions and editorial control remain with the researcher, and more stringent policies by journals for publication of industry-sponsored studies and scientific reviews. Ultimately, increased government and other independent support for nutrition research will diminish the attractiveness of industry funding to investigators and dilute any bias resulting from publication of industry-funded science.

## Abbreviations

CI: confidence interval
OR: odds ratio
